# Quantitative analysis of particles, genomes and infectious particles in supernatants of haemorrhagic fever virus cell cultures

**DOI:** 10.1186/1743-422X-8-81

**Published:** 2011-02-24

**Authors:** Manfred Weidmann, Amadou A Sall, Jean-Claude Manuguerra, Lamine Koivogui, Aime Adjami, Faye Fatou Traoré, Kjell-Olof Hedlund, Gunnel Lindegren, Ali Mirazimi

**Affiliations:** 1 Department of Virology, University Medical Center Göttingen, Germany; 2 l' Institut Pasteur de Dakar, Senegal; 3 Institut Pasteur, CIBU, Paris, France; 4 Institute of Medical Microbiology Université de Conakry, Guinea; 5 Multi Disease Surveillance Centre WHO, Ougadougou, Burkina Faso; 6 Fondation Merieux Mali, Bamako, Mali; 7 Center for microbiological preparedness,Swedish Institute for Infectious Disease Control, Solna, Sweden

## Abstract

Information on the replication of viral haemorrhagic fever viruses is not readily available and has never been analysed in a comparative approach. Here, we compared the cell culture growth characteristics of haemorrhagic fever viruses (HFV), of the *Arenaviridae*, *Filoviridae*, *Bunyaviridae*, and *Flavivridae *virus families by performing quantitative analysis of cell culture supernatants by (i) electron microscopy for the quantification of virus particles, (ii) quantitative real time PCR for the quantification of genomes, and (iii) determination of focus forming units by coating fluorescent antibodies to infected cell monolayers for the quantification of virus infectivity.

The comparative analysis revealed that filovirus and RVFV replication results in a surplus of genomes but varying degrees of packaging efficiency and infectious particles. More efficient replication and packaging was observed for Lassa virus, and Dengue virus resulting in a better yield of infectious particles while, YFV turned out to be most efficient with only 4 particles inducing one FFU. For Crimean-Congo haemorrhagic fever virus (CCHFV) a surplus of empty shells was observed with only one in 24 particles equipped with a genome. The complete particles turned out to be extraordinarily infectious.

## Background

Viral haemorrhagic fevers (VHF) are caused by various haemorrhagic fever viruses (HFV), of the *Arenaviridae*, *Filoviridae*, *Bunyaviridae*, and *Flavivridae *virus families. Only few laboratories specialize in the research on these agents. Basic virological information on these viruses is scant and described exclusively in the frame of case reports and pathological animal models. Although some progress has been achieved concerning the interaction of these viruses with mechanisms of innate immunity [1-9] and nitrite oxide pathways (CCHFV) [7] concise information on their basic virological characteristics is limited. This type of data however is important for biosafety risk assessment purposes. Here, we present a comparative analysis of quantities of HFV in cell culture determined by electron microscopic counting, quantitative real time RT-PCR and focus forming units, which reveal some features of the replication of these viruses that have not been described before.

## Methods

### Virus propagation

Viral stocks were prepared from Lassa virus (LASV) strain Josiah, Ebola Zaire virus (EBOVZ) strain Mayinga, Ebola Sudan virus (EBOSV) strains Maridi and Boniface, Marburg virus (MARV) strains Ravn, Ozolin and Musoke, Crimean Congo Haemorrhagic fever virus (CCHFV) strain IbAr 10200, Rift Valley fever virus (RVFV) strain ZH 548, Dengue 1 virus (DENV 1) strain 293, Yellow fever virus (YFV) strain Asibi. Confluent Vero cells (ATCC CCL-81) were inoculated with the respective virus and grown between 1 and 10 days in minimal essential medium (MEM) supplemented with penicillin/streptomycin solution, hepes and 2% heat inactivated foetal calf serum (all Gibco^®^BRL, Invitrogen, Life Technologies, Paisley, U.K.), at 37°C. The supernatants with progeny virus were collected and stored at -80°C until use. All handling of live virus was carried out in biosafety (BSL) level 3 or 4 facilities.

### Electron microscopy

The electron microscopy studies were performed using a Philips CM100 electron microscope (Eindhoven, The Netherlands) as previously described [10]. The viral supernatants were fixed in 2.5% glutaraldehyde (ratio 1:1) for a minimum of 1 hour in BSL3/4 laboratory, before decontamination of the tubes and transfer to BSL2 laboratory following safety instructions. After fixation, virus particles were pelleted on carbon/Formvar-coated 400-mesh copper grids (GilderGrids, Lincolnshire, England). Briefly, 150 μl of virus suspension was centrifuged for 10 min in an Eppendorf 5417C centrifuge (Hamburg, Germany) with a swing-out rotor at a maximum force of 12,000 × *g*. The grids were placed on the flat bottom of the outer container of Sarstedt microvette CB 300 tubes (Nümbrecht- Rommelsdorf, Germany). Ten grid squares were counted in each case. One particle per square equals a concentration of 1.5 × 10^5 ^particles per ml. Grids were stained by 2% tungstophosphoric acid (Merck, Darmstadt, Germany) at pH 6.

### Determination of focus-forming units (FFU)

The viruses were titrated 10-fold from 1:10 to 1: 10^10 ^in MEM and 100 μl of each dilution was transferred to confluent Vero cells in 96 well tissue culture plates, followed by incubation for 24-48 hours at 37°C and 5%CO_2_.

Subsequently the supernatants were removed, and the infected cells were washed 3 times with PBS, before fixation by cold acetone (80%) in distilled water for 60 minutes at -20°C. After fixation, fluorescent focus-forming assays were performed by incubation of specific antibodies on the infected cells for 30 minutes at 37°C, followed by incubation with secondary fluorescein-conjugated antibodies for additionally 30 minutes at 37°C (table 1). End point titres (FFU) were set at the last dilution giving unequivocal fluorescence.

**Table 1:** Description of antibodies used for determination of FFU

Virus	Primary antibodies	Secondary antibodies
LASV	Mouse serum Anti-Lassa virus (NP), (in-house)	FITC-conjugated Rabbit Anti-Mouse Immunoglobulins, (DAKO Cytomation, Denmark)
EBOZV/EBOSV	Mouse serum Anti-Ebola virus (NP), (in-house)	FITC-conjugated Goat Anti-Mouse IgG, (Jackson ImmunoResearch, Baltimore, USA)
MARV	Human patient serum	FITC-conjugated Goat Anti-Human IgG (FC specific), (Sigma-Aldrich Ltd., UK)
CCHFV	Rabbit serum Anti-CCHFV (NP), (in-house)	FITC-conjugated Goat Anti-Rabbit IgG (H+L), (Jackson ImmunoResearch, Baltimore, USA)
RVFV	Mouse polyclonal antibodies (provided by Michele Bouloy, Pasteur Institute, Paris).	FITC-conjugated Rabbit Anti-Mouse Immunoglobulins, (DAKO Cytomation, Denmark)
DENV 1	Mouse monoclonal to Dengue Virus E glycoprotein, (Abcam, Cambridge, UK)	FITC-conjugated Goat Anti-Mouse IgG, (Jackson ImmunoResearch, Baltimore, USA)
YFV	Mouse Monoclonal to Yellow Fever Virus, (Abcam, Cambridge, UK)	FITC-conjugated Goat Anti-Mouse IgG, (Jackson ImmunoResearch, Baltimore, USA)

### Quantitative real time PCR

RNA was isolated in a BSL3/4 laboratory from the viral stocks by treatment with TRIZOL Reagent (ratio 1+3) (Invitrogen) for a minimum of 5 minutes, before decontamination of the tubes and transport to a BSL2 laboratory following the safety instructions. Quantitative real time RT-PCR (qPCR) was performed using published primers and probes and RNA standards for RVFV, YFV, EBOZV, EBOSV, MARV and DENV [11-15] and primers and probes in table 2 for CCHFV and LASV. CCHFV primers were designed in reference to sequences U39455, AF467768, NC_005300, of African CCHFV isolates, the LASV primers were designed in reference to sequences J04324, AF333969 and AF246121. Quantitative RNA standards for CCHFV, LASV were transcribed from the M-segment (CCHFV), the nucleoprotein gene (LASV) and the 3' NTR region (DENV) ligated into pCRII, and evaluated as previously described [13]. qPCR was performed using the QuantiTect Probe RT-PCR Kit (Qiagen, Hilden Germany) on the Light Cycler 2.0 (Roche, Mannheim, Germany), and the following temperature protocol: RT 50°C for 5 min, activation at	 95°C for 15 min, amplification for 45 cycles at 95°C for 5 sec and 60°C for 15 sec. For CCHFV the same protocol with a touchdown in two degree steps from 70°C to 64°C for 3 cycles each and 33 cycles at 62°C was used.

**Table 2:** Primers and probes

Virus	Oligomer	Sequence 5`to 3'
CCHFV	CCFM FP	TCACCTTAGAGGAGGACACTGAAGG
	CCFM RP	CTCTTTTGAAAGAAAGTGTCATCACAATC
	CCHF M LNA	6FAM - TGGTGT**AA**G**A**G**AAA**TC - BBQ
LASV	LAS FP	YAACTCTGCATTYTTCACATCCC
	LAS RP	TGGGMAACCTAAGYTCACAGCA
	LAS P	6FAM - ACCACTCCATCTCTCCCAGCC - TMR

LNA Nucleotides in bold, IUB code used Y for C/T, M for A/C, dyes and quenchers used: 6FAM =, 6-Carboxyfluorescein BBQ = Black Berry Quencher, TMR = Tetramethylrhodamin

## Results

### Real time PCR

The new qPCR assays for the CCHFV 10200 M-segment, for LASV Josiah-N and DENV 3'NTR showed an analytical sensitivity of 100, 10 and 100 detected RNA molecules and efficiencies (E = 10^-1/slope^-1) of 0.9, 1.3 and 1.5 respectively.

### Determination of virus quantities

The virus titers were determined from 1 ml virus tissue culture supernatant by (i) electron microscopy (EM), (ii) quantitative real time PCR (qPCR) of RNA genomes extracted from the culture supernatant and (iii) determination of focus forming units (FFU) on cell monolayers infected with the supernatant using fluorescent antibodies.

The viral genome counts were generally from 1 to 2 orders of magnitude (log_10_-steps) higher than the particle counts (table 3, column 4). The FFU counts showed much greater differences being up to 5 log_10_-steps lower than the particle counts (table 3, column 5) and 1-6 log_10_-steps lower than genome counts (table 3, column 6).

**Table 3:** Quantitative detemination of virus titers

Virus	EM particles/ml	PCR genomes/ml	Infectivity FFU/ml	log_10 _difference PCR to EM	log_10 _difference FFU to EM	log_10 _difference FFU to PCR
EBOZV Mayinga	2.5 × 10^8^	8.3 × 10^10 ^± 3.2 × 10^9^	1.0 × 10^5^	+2	-3	-5
EBOSV Maridi	7.5 × 10^8^	8.6 × 10^10 ^± 5.4 × 10^9^	1.0 × 10^3^	+2	-5	-7
EBOSV Boniface	8.0 × 10^8^	5.9 × 10^10 ^± 3.4 × 10^9^	1.0 × 10^4^	+2	-4	-6
MARV Ravn	4.0 × 10^8^	1.3 × 10^8 ^± 1.8 × 10^6^	1.0 × 10^5^	0	-3	-3
MARV Ozolin	5.0 × 10^9^	1.6 × 10^9 ^± 1,1 × 10^8^	1.0 × 10^6^	0	-3	-3
MARV Musoke	3.0 × 10^9^	5.8 × 10^8 ^± 2.7 × 10^7^	1.0 × 10^5^	-1	-4	-3
LASV Josiah	1.0 × 10^6^	2.2 × 10^7 ^± 4.7 × 10^6^	1.0 × 10^4^	+1	-2	-3
RVFV ZH548	2.5 × 10^9^	2.9 × 10^11 ^± 4.3 × 10^9^	1.0 × 10^6^	+2	-3	-5
DENV-1 231	3.0 × 10^9^	4.6 × 10^10 ^± 4.3 × 10^9^	1.0 × 10^6^	+1	-3	-4
YFV Asibi	4.0 × 10^8^	8.2 × 10^9 ^± 1.7 × 10^8^	1.0 × 10^8^	+1	0	-1
CCHFV 10200	2.5 × 10^7^	1.0 × 10^6 ^± 6.1 × 10^5^	1.0 × 10^6^	+1	-1	-2

EM: calculated from 10 grid squares, qPCR: average and standard deviation of triplicate qPCR results (8 qPCR results for CCHFV), FFU: mean of three titrations.

The EBOZV and EBOSV strains showed a genome count +2 log_10_-step higher than the particle count, but much lower FFU counts differing by -3 to -5 log_10_-steps from the particle count. MARV strains Ravn and Ozolin showed almost equal particle and genome counts, whereas MARV Musoke showed a -1 log_10_-step reduction in the genome count compared to the particle count. As for EBOZV and EBOSV, the FFU counts of the MARV strains were -3 to -4 log_10_-steps lower than the particle counts.

The difference between genome and particle count for LASV, RVFV, and DENV was between +1 to +2 log_10_-steps. The FFU reduction from the particle count for these viruses ranged between -2 and -3 log_10_-steps.

YFV was the only virus were particle and FFU count equalled each other topped only by a +1 log_10_-step genome count and the lowest difference (-1 log_10_-step) of the FFU count to the genome count.

Apart from YFV, CCHFV showed the lowest level of difference between FFU and particle count (-1 log_10_-step). In comparison to YFV a +1 log_10_-step increased genome count over the particle therefore leads to a -2 log_10_-step reduction of the FFU count to the genome count.

To interpret the data obtained, ratios were formed between all three quantitative data sets. The observed ratios of particles/FFU (table 4, column 1) point out that for LASV, CCHFV and YFV only few particles (1-10^2^) are associated with one FFU, whereas for all other viruses much higher amounts (10^3^-10^5^) were counted (10^3 ^for EBOZV, MARV Ravn, MARV Ozolin, RVFV and DENV 1, 10^4 ^for EBOSV Boniface, MARV Musoke and 10^5 ^for EBOSV Maridi).

**Table 4:** Selected ratios of quantitative titer results

Virus	particles/FFU	genomes/particle	particles/genome	genome/FFU
EBOZV Mayinga	2.5 × 10^3^	336	0.00	8.3 × 10^5^
EBOSV Maridi	7.5 × 10^5^	115	0.01	8.6 × 10^7^
EBOSV Boniface	8.0 × 10^4^	74	0.01	5.9 × 10^6^
MARV Ravn	4.0 × 10^3^	0.3	3.08	1.3 × 10^3^
MARV Ozolin	5.0 × 10^3^	0.3	2.98	1.6 × 10^3^
MARV Musoke	3.0 × 10^4^	0.2	5.10	5.8 × 10^3^
LASV Josiah	100	22	0.05	2.2 × 10^3^
RVFV ZH548	2.5 × 10^3^	119	0.01	2.9 × 10^7^
DENV 1 231	3.0 × 10^3^	15	0.06	4.6 × 10^4^
YFV Asibi	4	21	0.05	83
CCHFV IbAR 10200	25	0.04	24	1

The ratios of genomes per particle and its reciprocal (table 4, column 2 and 3) indicate that most viruses appear to be overproducing genomes only packaging a fraction of genomes into shells to create complete particles. EBOZV, EBOSV, and RVFV seem to produce a surplus in the range of 74 - 336 genomes in relation to the shells produced while this surplus is of reduced magnitude for LASV, DENV and YFV (about 15-20). MARV appears to be efficiently packaging every 3^rd ^- 5^th ^genome into a particle (table 4, column 3) achieving this by a slight overproduction of shells. The CCHFV machinery produces 23 empty shells for 1 particle packed with a genome. The packed genomes however are highly infectious as indicated by the value of 1 for the ratio of FFU/genomes (table 4 column 4).

## Discussion

The comparative quantitative analysis of virus titers in cell culture using three independent methods revealed some insight into the packaging efficiency of the viruses analysed. The viral RNA genomes were quantified from cell culture supernatants, which most certainly contained free RNA genomes from lysed cells for all of the viruses except for CCHFV, which does not induce a cytopathic effect in Vero cells. Nevertheless, there are some prominent differences in the magnitude of genomes and particle production observed.

In the group of the filoviruses there seems to be a clear separation between the growth characteristics of EBOZV and EBOSV on the one hand, and MARV strains on the other hand. While Ebola viruses appear to produce a 2 log_10_-step surplus of genomes over the particles detectable in EM, the particle and genome values for MARV are almost at the same level. Surprisingly in spite of the production surplus of 10^8^-10^9 ^particles both filovirus types seem to produce comparatively few infectious particles with FFU counts down by 3 to 4 log_10_-steps from the amount of particles (and genomes) for MARV and 4 to 5 log_10_-steps down for EBOZV and EBOSV. Due to the overproduction the resulting amounts of FFU are still veritable but it appears that efficient genome packing by MARV (1 genome in 3-5 particles) by producing more shells than genomes does not lead to a high rate of infectious particles, as about 1000 particles are needed to induce one FFU. For EBOZV and EBOSV a 100-fold overproduction of genomes only yields an even lower amount of infectious particles, as 10^3^-10^5 ^are needed to induce one FFU. It seems fair to say that at least as observed in cell culture particle and genome production seem to run out of sync resulting in a proportionally low amount of infectious particles.

In experimental animal models for EBOZV doses as low as one plaque-forming unit suffice to cause infection [16]. The amount of particles recorded for one FFU indicate that although particle production is not very efficient indeed the overall high amount of particles present in one plaque guarantee infection.

Cellular growth of RVFV seems comparable to that of EBOZV Mayinga with very high yields of particles (10^8^) and genomes (10^11^) leading to 1 genome in 119 being packed into a particle of which 10^3 ^are needed to induce one FFU. In recent experiments sheep were successfully inoculated with RVFV at a dose of 1 × 10^5 ^TCID_50 _[17]. TCID_50 _and FFU are not easy to relate but it seems that there is much scope to determine a much lower infectious dose of RVFV.

The difference between particle count and genome count for LASV is low and reflected in a genome to particle ratio of 22 indicating a good correlation between virus particles and genomes produced. The efficiency of the virus particles and their infectiousness (100/FFU) observed in cell culture is reflected by the fact that animal models can be highly susceptible to lethal infection as for example to 2 PFU for inbred strain 13 guinea pig [18]. A similar efficiency of the LASV particles was observed in LASV virus-infected marmoset tissues, which contained significant amounts of viral RNA comparable with levels of viremia (measured in PFU) [19].

The best yield of correctly packaged particles is obtained by YFV and CCHFV indicating efficient synchronisation of shell and genome production (table 4, column 4) by these viruses.

DENV packs genomes even more efficiently than LASV and YFV (1 in 15 as compared to 1 in 20). The resulting DENV particles are however much less infectious than the YFV particles and about 10^3 ^are needed to induce one FFU.

YFV presents itself as the most efficient packer, leading to a very high ratio of infectious particles since it is the only virus for which particle count and FFU count are the same. From the ratios formed it appears that a slight overproduction of genomes (1 log_10 _- steps up from the particle count) is conducive to a genome packaging ratio of 1 in 21 of which only 4 are needed to induce one FFU. Rhesus monkeys have been successfully infected with 7 × 10^2 ^plaque forming units (PFU) determined on vero E6 cells [20]. Our results show that the YFV particles indeed are highly infectious and that the infectious dose needed for animal models might be even below the dose used.

**Figure 1:**
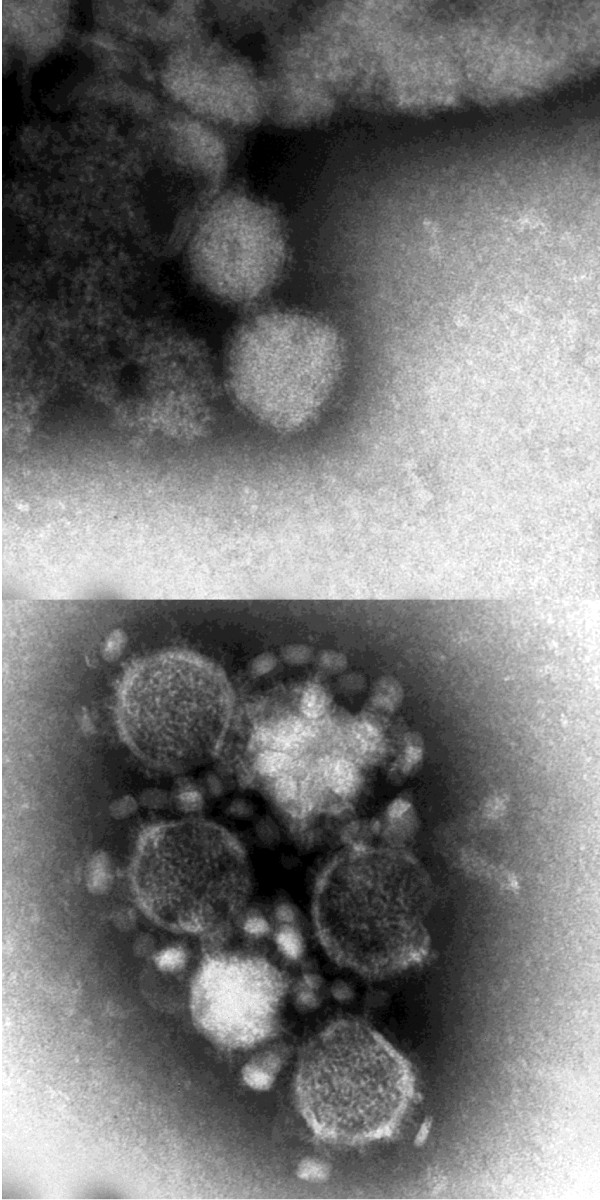
**Electron microscopic pictures of malformed CCHFV particles**. Upper panel particles without glycoproteins in lipid membrane, lower panel: mixture of whole particles with glycoproteins and malformed particles without glycoproteins.

CCHFV does not induce a CPE in vero cells and therefore additional viral RNA genomes released by cell lysis do not contribute to the genome count. Therefore the assembly modalities of the infectious CCHFV particles appear reverse to those of the other viruses, as only every 24th particle will actually contain a genome leading to a surplus of empty shells. The correctly assembled particles however are highly infectious as each of them induces a FFU. In the EM analysis malformed CCHFV particles were noticed (see Figure 1). Defective interfering particles (DIP) have been described for almost all types of DNA and RNA viruses. DIP have an influence on the viable virus yields (measured in PFU) in cell culture. Particular ratios of DIP to viable virus are conducive to good or bad yields of progeny virus. The mathematical models for this relationship have been extended recently [21]. The extraordinary infectious CCHFV particles (1 genome/FFU) indicate that the malformed CCHFV particles observed might be DIP driving this type of yield.

A recent study showed that 10 FFU sufficed to kill IFNAR ^-/- ^mice lacking the IFN receptor. These mice do not develop an innate immune response and therefore develop acute disease which makes them a good model for CCHFV infection and disease [22]. These results confirm the extraordinary infectiousness of the CCHFV particles observed in our study.

## Conclusion

The analysed quantitative virus titers indicate that YFV and CCHFV virus are the most efficient in producing infectious particles. These viruses manage to synchronise genome and particle production in an optimal fashion as opposed for example to the filoviruses with an apparent overflowing and inefficient production of genomes and particles. YFV and CCHFV represent the optimal synchronisation of the replication strategies observed i.e. overproduction of genomes and overproduction of shells respectively.

## Competing interests

The authors declare that they have no competing interests.

## Authors' contributions

MW: performed qPCR drafted manuscript, AS: provided strains, involved in drafting and revising the manuscript, has given final approval of the version to be published.

JCM: involved in drafting and revising the manuscript, has given final approval of the version to be published, LK: revised the manuscript, has given final approval of the version to be published, AA: revised the manuscript, has given final approval of the version to be published, FFT: revised the manuscript, has given final approval of the version to be published, KOH: performed EM, has given final approval of the version to be published, GL: performed FFU assay, has given final approval of the version to be published, AM: involved in drafting and revising the manuscript, has given final approval of the version to be published.
